# Supplementary material for: Double-Clad Antiresonant Hollow-Core Fiber and Its Comparison with Other Fibers for Multiphoton Micro-Endoscopy
Source: Sensors (Basel). 2024 Apr 12;24(8):2482. doi: 10.3390/s24082482 (PMC11054428; doi:10.3390/s24082482)
Supplement: Supplementary file 1 [file sensors-24-02482-s001.zip › sensors-2852250-supplementary.pdf]

Supplementary information

Double Clad Antiresonant Hollow Core Fiber and Its Comparison with other Fibres for Multiphoton Micro-Endoscopy

Marzanna Szwaj[1,2,3] , Ian A Davidson[1], Peter Johnson[2,3], Gregory Jasion[1], Yongmin Jung[1], Seyed Reza Sandoghchi [1], Krzysztof Herdzik[1,2,3], Konstantinos Bourdakos[2,3], Natalie V. Wheeler[1], Hans Christian Mulvad[1], David J Richardson[1], Francesco Poletti[1*], Sumeet Mahajan[2,3]*

[1]*Optoelectronics Research Centre, University of Southampton, SO17 1BJ, UK*
[2]*Institute for Life Sciences, University of Southampton, SO17 1BJ, UK*
[3]*School of Chemistry, University of Southampton, SO17 1BJ, UK*
**frap@orc.soton.ac.uk*
**S.Mahajan@soton.ac.uk*

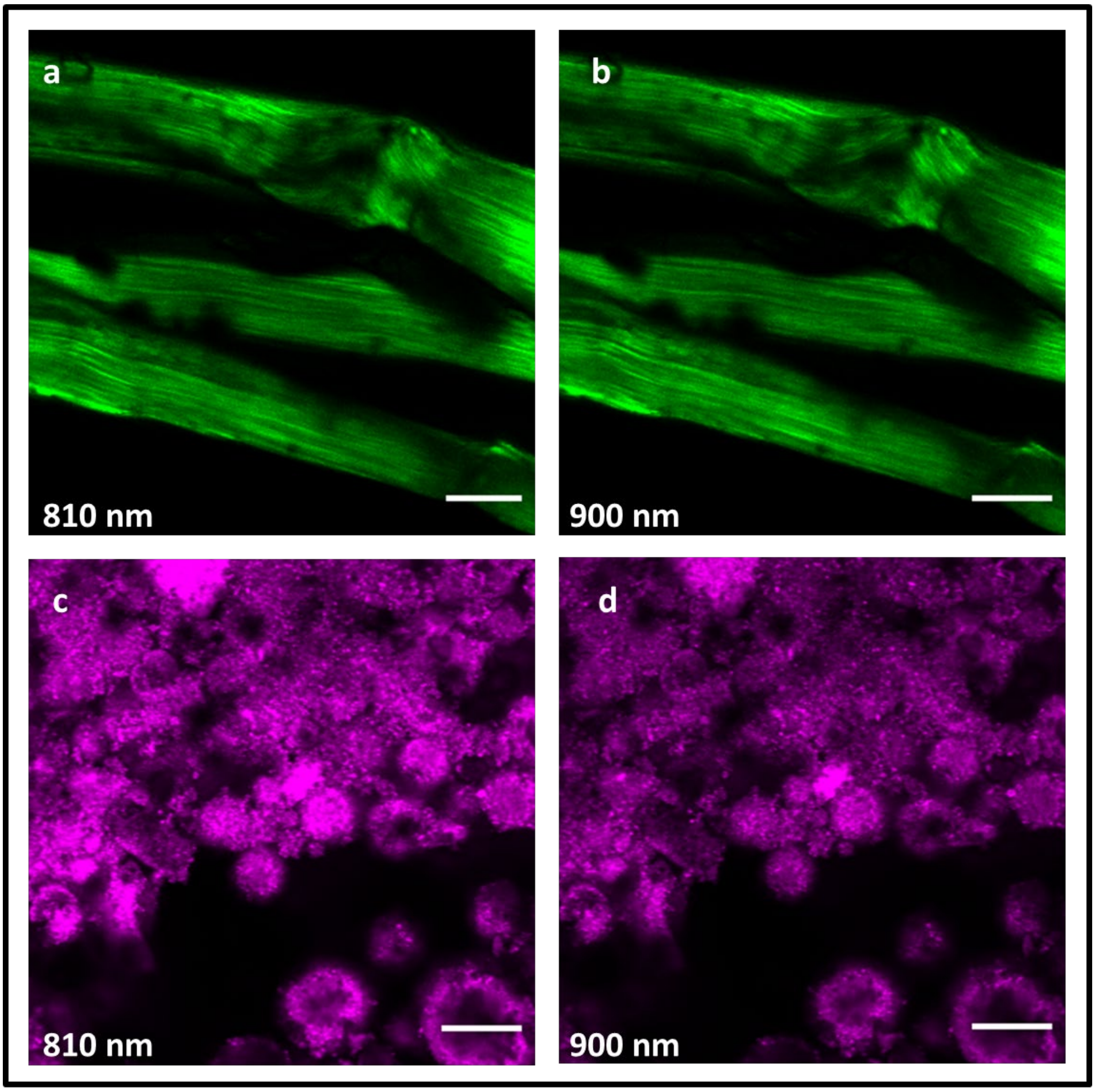

Fig. S1 Mouse tail tendon and barium titanate nano-crystals images using free-space laser propagation. The images of mouse tail tendon (a, b) and barium titanate crystal (c, d) captured using a free-space laser propagation at 810 nm and 900 nm excitation wavelength (20x objective, zoom 3, 341 pixels x 341 pixels, 10.7 µs dwell time, ~ 20 mW power). Scale bar = 50 µm.

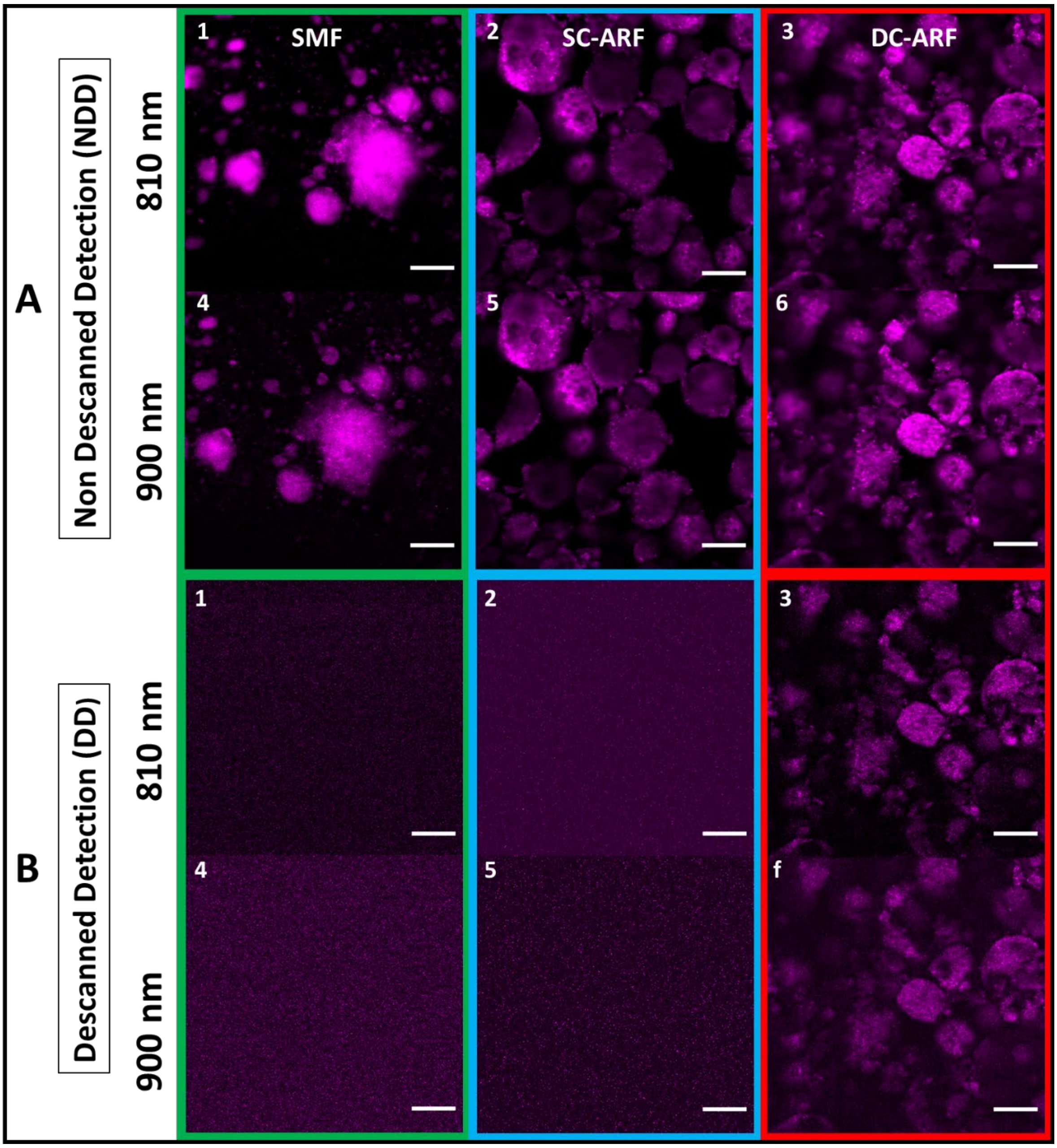

Fig. S2 SHG images of Barium titanate nano-crystals with all fibers in non-descanned and descanned configurations. The images of barium titanate crystal taken using NDD **A (1-6)** and DD **B (1-6)** configuration 1 meter long SCF, SC-ARF and DC-ARF at 810 nm and 900 nm excitation wavelength (NDD: 20x objective, zoom 3, 341 pixels x 341 pixels, 10.7 µs dwell time, ~20 mW power; DD: 20x objective, zoom 3, 341 pixels, 27 µs dwell time, ~ 20 mW power, average 10 frames). Scale bar = 50 µm.

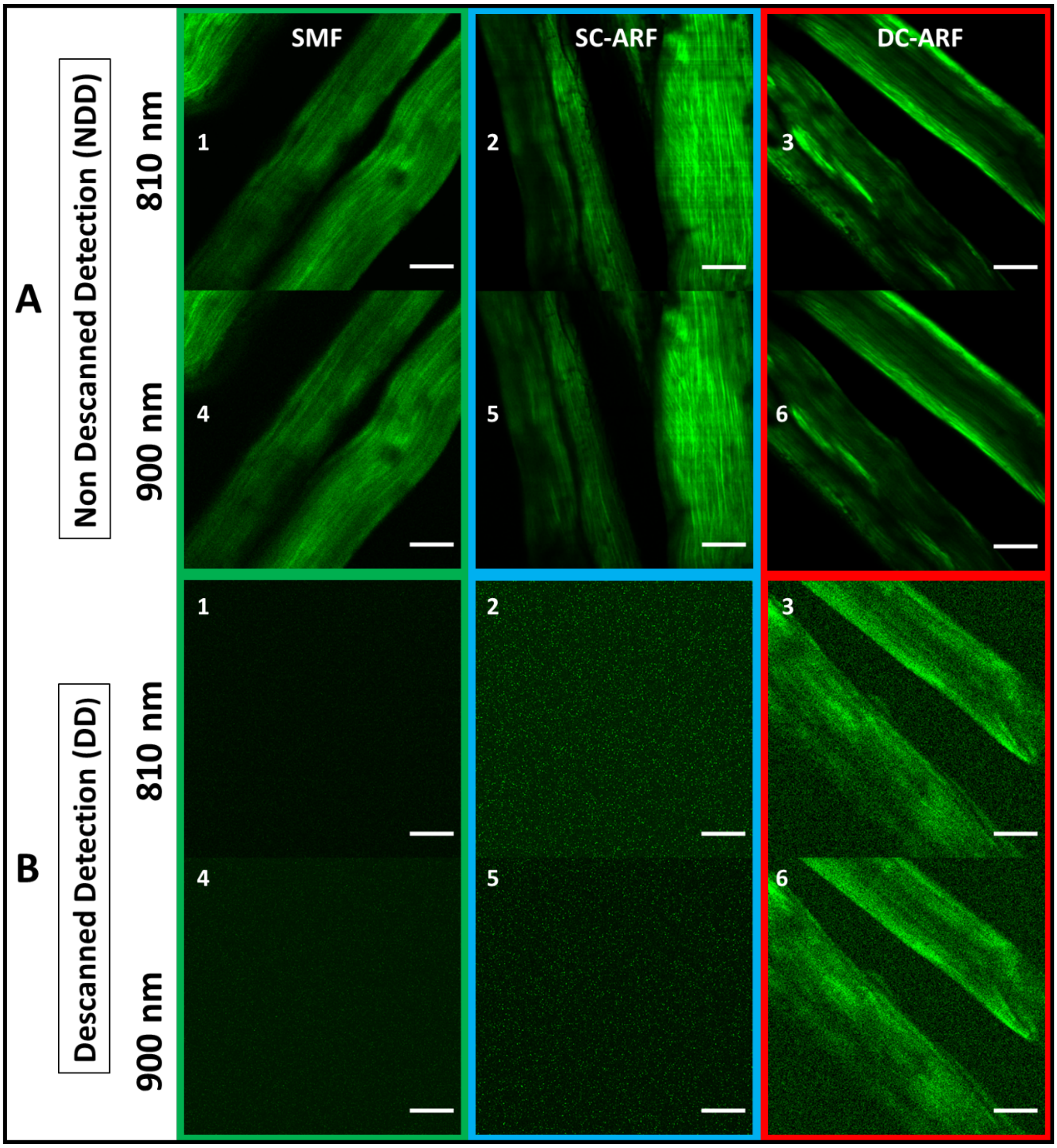

Fig. S3 SHG images of mouse tail tendon with all fibers of 1 m length in non-descanned and descanned configurations. The images of mouse tail tendon taken using NDD **A (1-6)** and DD **B (1-6)** configuration (with their schematic representation) for 1 meter long SCF, SC-ARF and DC-ARF at 810 nm and 900 nm excitation wavelength (NDD: 20x objective, zoom 3, 341 pixels x 341 pixels, 10.7 µs dwell time, ~20 mW power; DD: 20x objective, zoom 3, 341 pixels x 341 pixels, 27 µs dwell time, ~20 mW power, average 10 frames). Scale bar = 50 µm.

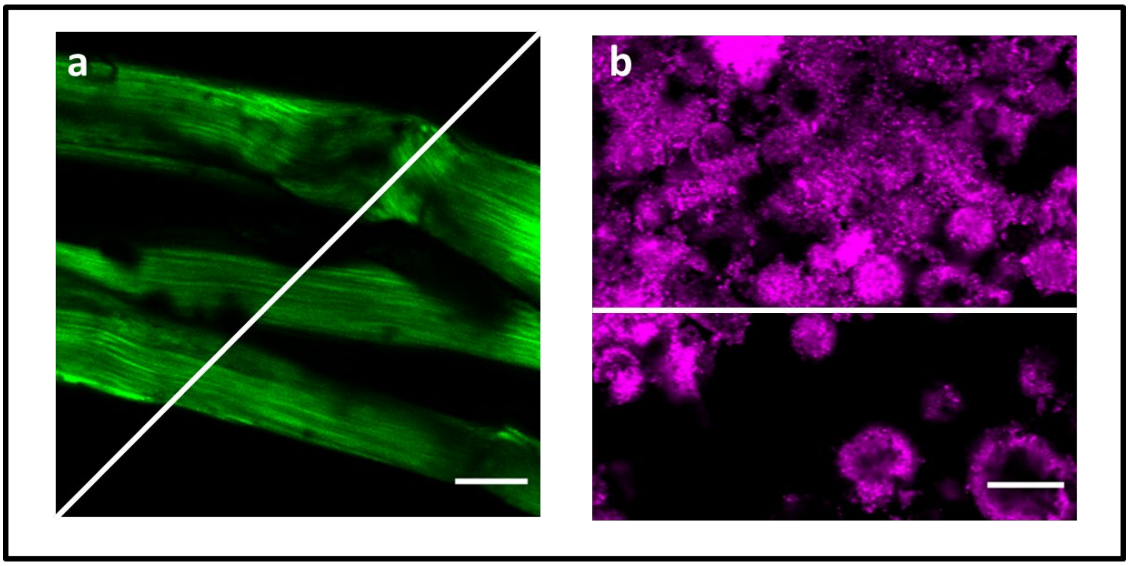

Fig. S4 Line profile examples. Example of mouse tail **(a)** and barium titanate crystal **(b)** images with the line profiles used in **Fig. 5** and **S4** respectively. Line profiles were taken across areas that had both the sample as well as some blank areas.

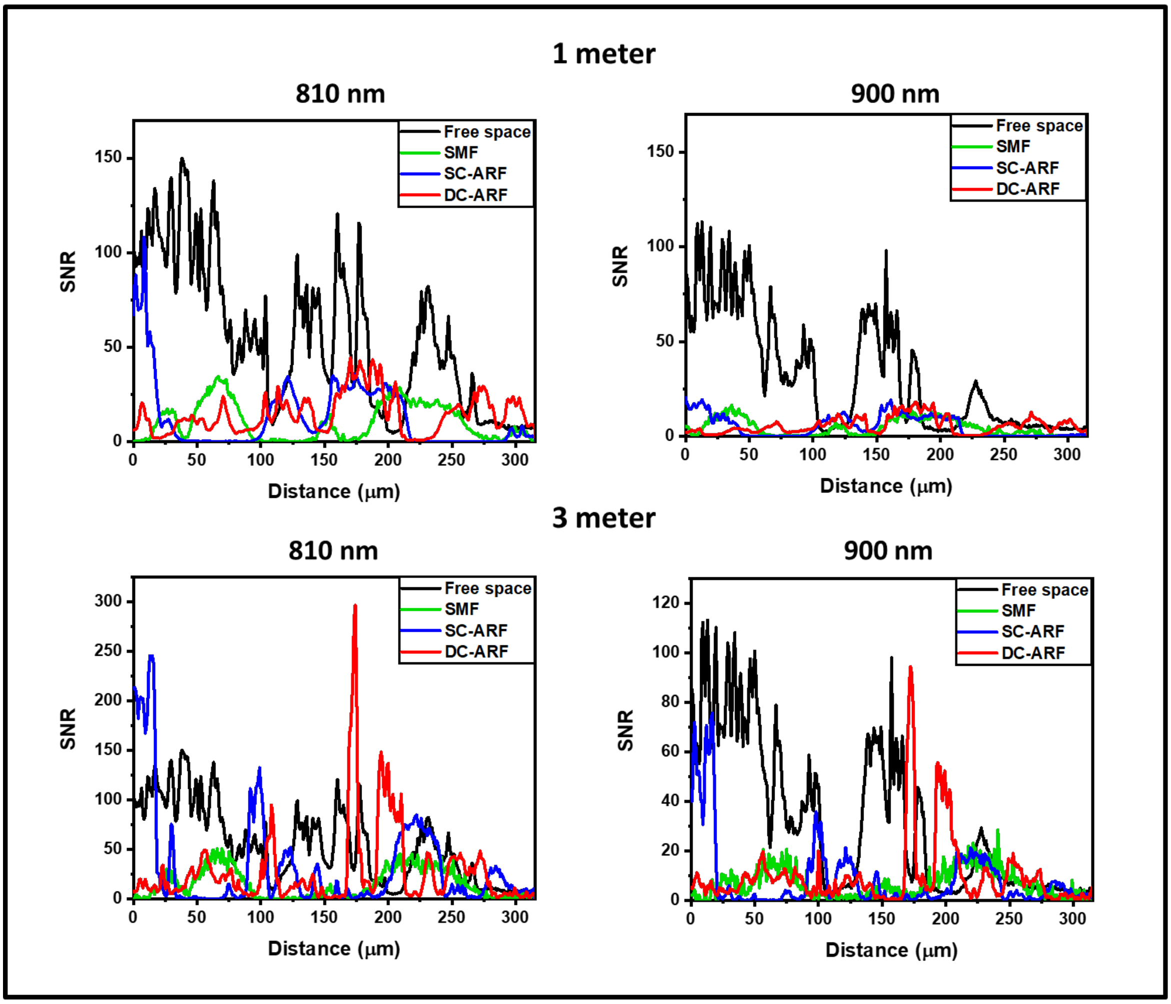

Fig. S5 Average SNR vs distance plots. These profiles were obtained for 1 and 3 m SCF, SC-ARF, and DC-ARF in NDD compared with the imaging configuration with a free-space laser coupled. These SNR profiles are calculated for images of a barium titanate nanocrystal sample.

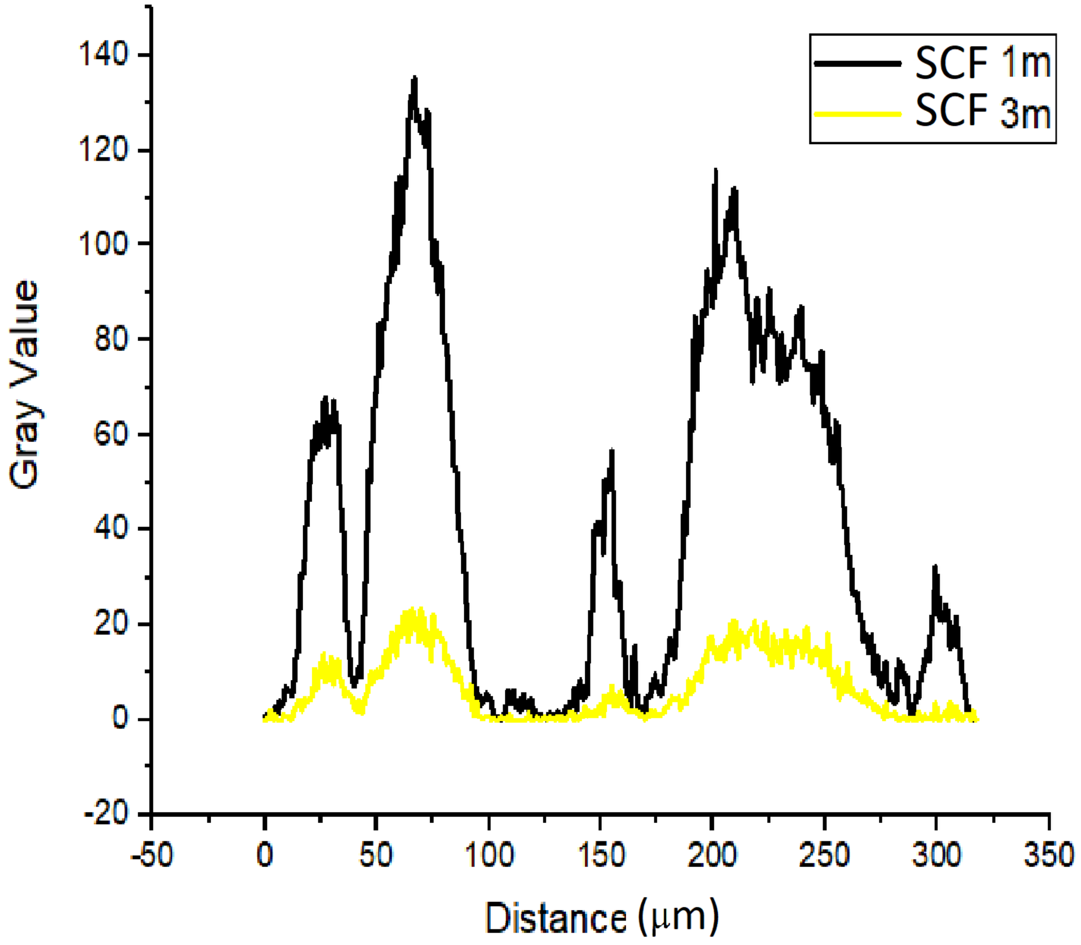

Fig. S6 Signal profiles for SCF. Signal intensities over a line profile of the same image for a 1 and a 3 m SCF. The signals decrease approximately 7 times due to anomalous GVD and non-linearity. The images were collected in the NDD configuration.

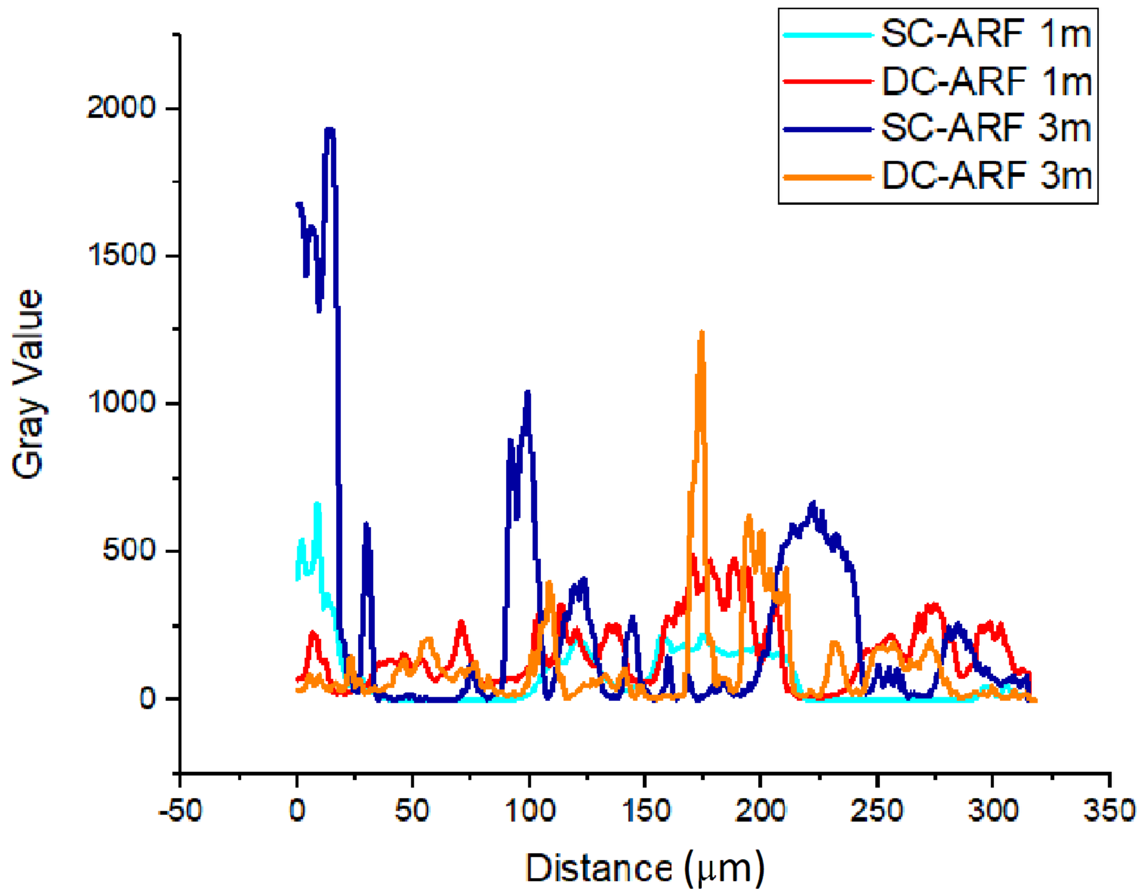

Fig. S7 Signal profiles for SC-ARF and DC-ARF. Signal intensities over a line profile of approximately the same imaging area (due to alignment issues the image was not exactly the same) for a 1 and a 3 m SC-ARF and DC-ARF. The signals are of approximately similar levels between the two lengths in either of the fibers. The images were collected in the NDD configuration.
